# Supplementary material for: Qigong Therapy for Stress Management: A Systematic Review of Randomized Controlled Trials
Source: Healthcare (Basel). 2024 Nov 23;12(23):2342. doi: 10.3390/healthcare12232342 (PMC11641396; doi:10.3390/healthcare12232342)
Supplement: Supplementary file 1 [file healthcare-12-02342-s001.zip › Additional File S2. Search terms used in each database and results.pdf]

Supplement S1. Search terms used in each database and results

Medline via PubMed (14.10.2024.)

	Searches
#1	qigong[TW] OR “qi gong”[TW] OR “qi-gong”[TW] OR “chi gong”[TW] OR “chi kung”[TW] OR “chi chung”[TW] OR qi-training[TW]
#2	Stress[TW]
#3	“randomized controlled trial”[PT] OR “controlled clinical trial”[PT] OR randomized[TIAB] OR placebo[TIAB] OR “drug therapy”[SH] OR randomly[TIAB] OR trial[TIAB] OR groups[TIAB]
#4	animals[MH] NOT humans[MH]
#5	#1 AND #2 AND #3 AND #4 NOT #5

Embase via Elsevier (15.10.2024.)

	Searches
#1	qigong/exp OR qigong:ab,ti OR ‘qi gong’/exp OR ‘qi gong’:ab,ti OR qi-gong/exp OR qi-gong:ab,ti OR ‘chi gong’/exp OR ‘chi gong’:ab,ti OR ‘chi kung’/exp OR ‘chi kung’:ab,ti OR ‘chi chung’/exp OR ‘chi chung’:ab,ti OR qi-training/exp OR qi-training:ab,ti
#2	stress/exp OR stress:ab,ti
#3	'crossover procedure':de OR 'double-blind procedure':de OR 'randomized controlled trial':de OR 'single-blind procedure':de OR (random* OR factorial* OR crossover* OR cross NEXT/1 over* OR placebo* OR doubl* NEAR/1 blind* OR singl* NEAR/1 blind* OR assign* OR allocat* OR volunteer*):de,ab,ti
#4	#1 AND #2 AND #3

PEDro (15.10.2024.)

	Searches
#1	qigong
#2	qi gong
#3	qi-gong
#4	chi gong
#5	chi kung
#6	chi chung
#7	qi-training
#8	#1 OR #2 OR #3 OR #4 OR #5 OR #6 OR #7
#9	stress
#10	randomized controlled trial
#11	randomised controlled trial
#12	#10 OR #12
#13	#8 AND #9 AND #12

CENTRAL (23.10.2024.)

	Searches
#1	MeSH descriptor: [Qigong] explode all trees
#2	(qigong OR (qi gong) OR qi-gong OR (chi gong) OR (chi kung) OR (chi chung) OR qi-training):ti,ab,kw
#3	#1 OR #2
#4	MeSH descriptor: [Stress, Psychological] explode all trees
#5	Stress:ti,ab,kw
#6	#4 OR #5
**#7**	**(#3 AND # 6) in Trials**

CINAHL (EBSCOhost) (23.10.2024.)

	Searches
#1	MH “qigong”
#2	TX "qigong" OR “qi gong” OR “qi-gong” OR “chi gong” OR “chi kung” OR “chi chung” OR “qi-training”
#3	#1 OR #2
#4	MH “stress”
#5	TX "stress"
#6	#4 OR #5
#7	MH "Clinical Trials"
#8	TX “trial” OR "single-blind*" OR "double-blind*" OR "treatment as usual" OR “randomly”
#9	#7 OR #8
**#10**	**#3 AND #6 AND #9**

Korea Institute of Science and Technology Information (ScienceON) (23.10.2024.)

	Searches
#1	“qigong” OR “qi gong” OR “qi-gong” OR “chi gong” OR “chi kung” OR "chi chung" OR "qi-training"
#2	"stress"
#3	“clinical trial” OR “randomized controlled trial” OR “randomised controlled trial”
#4	#1 AND #2 AND #3

Korean traditional knowledge portal (23.10.2024.)

	Searches
#1	(기공) &(스트레스) & (무작위|임상)

KoreaMed (23.10.2024.)

	Searches
#1	(qigong[ALL] OR qi gong[ALL] OR qi-gong[ALL] OR chi gong[ALL] OR chi kung[ALL] OR chi chung[ALL] OR qi-training[ALL]) AND stress[ALL] AND (clinical trial[ALL] OR randomized controlled trial[ALL] OR randomised controlled trial[ALL])

OASIS (23.10.2024.)

	Searches
#1	qigong|qi gong| chi gong|chi kung|chi chung|qi training
#2	Stress
#3	random|clinical trials
#4	#1 AND #2 AND #3

RISS (23.10.2024.)

	Searches
#1	qigong|qi gong| chi gong|chi kung|chi chung|qi training
#2	Stress
#3	random|clinical trials
#4	#1 AND #2 AND #3

National Library of Korea (23.10.2024.)

	Searches
#1	qigong
#2	stress
#3	random
#4	#1 AND #2 AND #3
