# Supplementary material for: Qigong Therapy for Stress Management: A Systematic Review of Randomized Controlled Trials
Source: Healthcare (Basel). 2024 Nov 23;12(23):2342. doi: 10.3390/healthcare12232342 (PMC11641396; doi:10.3390/healthcare12232342)
Supplement: Supplementary file 1 [file healthcare-12-02342-s001.zip › Additional File S3. Details of Qigong program.pdf]

Supplement S3. Details of Qigong program

First author (Year) country	Details of Qigong program
Griffith JM (2008) [23]	The Qigong exercises employed were The Basic Eight, a medical Qigong set consisting of 8 exercises designed by Qigong grandmaster Hong Liu to activate 14 of the meridians<br>1. Lung and large intestine<br>2. Stomach and Spleen<br>3. Heart and Small Intestine<br>4. Bladder and Kidney<br>5. Pericardium and Triple Heater<br>6. Gallbladder and Liver<br>7. Conception Vessel<br>8. Governing Vessel
Hwang EY (2010) [24]	1. Relaxed breathing,<br>2. Relaxing the body<br>3. Gathering Qi and self-healing.
Chow YWY (2012) [25]	Each of them has solid experience in Qigong, other kinds of mindful exercise orphysical exercise for body–mind well-being in his/her own profession.<br>Details of program were not reported
Chan ES (2013) [26]	1. Warm-up<br>2. Qigong exercise(45min)<br>(1) tensing and relaxing of the hands<br>(2) standing still<br>(3) scanning front part of the body with palms<br>(4) gentle bending and stretching of the spine<br>3. Cooling-down stage.
Hwang EY (2013) [27]	1. relaxed breathing,<br>2. relaxing the body<br>3. gathering Qi and self-healing.
Cheung DST (2019) [28]	1-6 weeks: group training<br>7-22 weeks: weekly group follow-up

	It consists of 8 movements that are performed in a smooth and graceful manner.<br><br>The movements, combined with breathing and meditation, exercise the mind and body for healing.
Ng SM<br><br>(2022) [29]	1. Basic Traditional Chinese medicine and Qigong theories<br><br>2. 10 simple Qigong movements
Zhang S<br><br>(2022) [30]	1. Warm-up(5m)<br><br>2. Qigong Practice(30m)<br><br>(Action1) The subject slowly squats and puts his palms together in front of his chest. While inhaling, he slightly flexes the knee joints, keeping the knees not exceeding the toes as a suitable squatting angle. Keep the eyes on the ground about 3m in front of your body. Concentrate and maintain the above position for 10s. Then exhale while slowly straightening the knee joint. During this process, the eyes are closed tightly, and the participant feels the position of the knee joint. The operations at this stage also lasts for 10s.<br><br>(Action 2) The subject adopts a posture of standing back and forth with both feet, with the toes of the front feet on the ground, and the center of gravity on the other foot that is back. Place one hand 30cm above the side of the head, and place the other hand on the lumbosacral area, and then perform knee flexion and extension with inhalation and exhalation. The rhythm is the same as Action 1. Keep his eyes on the fingertips when f lexing the knees, and close his eyes when extending the knees which can focus on feeling the position of the knee joint.<br><br>(Action 3) The subject stand upright, raised his arms forward and inhaled, his heels slowly lifted off the ground, and the toes were held on the ground. While extending the knees, the subject looked at the ground about three meters in front and below, stabilized his center of gravity, maintained balance for 10s; then, while flexing the upper arm, the heel slowly drops, and he also need to feel the position of the knee joint.<br><br>(Action 4) Stand with the lower limbs separated by a distance of 1m, and extend the upper limbs to the limit in the horizontal direction. Then make a lunge with both lower limbs in the left and right directions, with the center of gravity on the forefoot, while inhaling, staring at the floor 3m ahead for 8–10s. Then the hip joint of the front lower limb is externally rotated, the knee joint is extended, and the knee joint of the rear lower limb is flexed, the center of gravity is on the hind foot, and the exhale is also maintained for 10s, and finally restored to the initial preparation stage posture.<br><br>(Action 5) Put his hands in front of the chest, and then flex his knee joint with inhalation (the range of flexion angle is larger than any other movement), put hands on the outside of the knee joint, holding for 2s, and keep the eyes on the ground 3m ahead; Then exhale, straighten the knee joint, close the eyes, feeling the position of the knee joint.<br><br>3. Cool-down(5m)
Wu F (2024) [31]	1. Meditation practice (10m)<br><br>2. Traditional Chinese medicine knowledge, breathing regulation knowledge (40m)

	3. Relaxation practice (5m)
	4. Q&A and homework assignments (5m)
