# Supplementary material for: Qigong Therapy for Stress Management: A Systematic Review of Randomized Controlled Trials
Source: Healthcare (Basel). 2024 Nov 23;12(23):2342. doi: 10.3390/healthcare12232342 (PMC11641396; doi:10.3390/healthcare12232342)
Supplement: Supplementary file 1 [file healthcare-12-02342-s001.zip › Additional File S4. Risk of Bias in the included studies.pdf]

Supplement 4. Risk of Bias in the included studies

[23] Griffith JM et al. Qigong stress reduction in hospital staff: a randomized controlled trial

**Domain**	**Signalling questions**	**Response options**
Domain 1: Risk of bias arising from the randomization process	1.1 Was the allocation sequence random?	Y
	1.2 Was the allocation sequence concealed until participants were enrolled and assigned to interventions?	NI
	1.3 Did baseline differences between intervention groups suggest a problem with the randomization process?	N
Domain 2: Risk of bias due to deviations from the intended interventions (effect of assignment to intervention)	2.1. Were participants aware of their assigned intervention during the trial?	PY
	2.2. Were carers and people delivering the interventions aware of participants's assigned intervention during the trial?	PY
	2.3. If Y/PY/NI to 2.1 or 2.2: Were there deviations from the intended intervention that arose because of the trial context?	Y
	2.4 If Y/PY to 2.3: Were these deviations likely to have affected the outcome?	Y
	2.5. If Y/PY/NI to 2.4: Were these deviations from intended intervention balanced between groups?	PN
	2.6 Was an appropriate analysis used to estimate the effect of assignment to intervention?	PY
	2.7 If N/PN/NI to 2.6: Was there potential for a substantial impact (on the result) of the failure to analyse participants in the group to which they were randomized?	NA
Domain 3: Missing outcome data	3.1 Were data for this outcome available for all, or nearly all, participants randomized?	N
	3.2 If N/PN/NI to 3.1: Is there evidence that the result was not biased by missing outcome data?	N
	3.3 If N/PN to 3.2: Could missingness in the outcome depend on its true value?	PY
	3.4 If Y/PY/NI to 3.3: Is it likely that missingness in the outcome depended on its true value?	PY
Domain 4: Risk of bias in measurement of the outcome	4.1 Was the method of measuring the outcome inappropriate?	N
	4.2 Could measurement or ascertainment of the outcome have differed between intervention groups?	N
	4.3 If N/PN/NI to 4.1 and 4.2: Were outcome assessors aware of the intervention received by study participants?	NI
	4.4 If Y/PY/NI to 4.3: Could assessment of the outcome have been influenced by knowledge of intervention received?	NI

	4.5 If Y/PY/NI to 4.4: Is it likely that assessment of the outcome was influenced by knowledge of intervention received?	PN
Domain 5: Risk of bias in selection of the reported result	5.1 Were the data that produced this result analysed in accordance with a pre-specified analysis plan that was finalized before unblinded outcome data were available for analysis?	PY
	5.2. ... multiple eligible outcome measurements (e.g. scales, definitions, time points)	PY
	5.3 ... multiple eligible analyses of the data?	PY

[24] Hwang EY et al. The correlation between the effectiveness of Brief Qigong-based Stress Reduction Program(BQSRP) and personality

**Domain**	**Signalling questions**	**Response options**
Domain 1: Risk of bias arising from the randomization process	1.1 Was the allocation sequence random?	Y
	1.2 Was the allocation sequence concealed until participants were enrolled and assigned to interventions?	NI
	1.3 Did baseline differences between intervention groups suggest a problem with the randomization process?	NI
Domain 2: Risk of bias due to deviations from the intended interventions (effect of assignment to intervention)	2.1. Were participants aware of their assigned intervention during the trial?	PY
	2.2. Were carers and people delivering the interventions aware of participants's assigned intervention during the trial?	PY
	2.3. If Y/PY/NI to 2.1 or 2.2: Were there deviations from the intended intervention that arose because of the trial context?	Y
	2.4 If Y/PY to 2.3: Were these deviations likely to have affected the outcome?	Y
	2.5. If Y/PY/NI to 2.4: Were these deviations from intended intervention balanced between groups?	Y
	2.6 Was an appropriate analysis used to estimate the effect of assignment to intervention?	PY
	2.7 If N/PN/NI to 2.6: Was there potential for a substantial impact (on the result) of the failure to analyse participants in the group to which they were randomized?	NA
Domain 3: Missing outcome data	3.1 Were data for this outcome available for all, or nearly all, participants randomized?	N
	3.2 If N/PN/NI to 3.1: Is there evidence that the result was not biased by missing outcome data?	N
	3.3 If N/PN to 3.2: Could missingness in the outcome depend on its true value?	PY
	3.4 If Y/PY/NI to 3.3: Is it likely that missingness in the outcome depended on its true value?	PN
Domain 4: Risk of bias in measurement of the outcome	4.1 Was the method of measuring the outcome inappropriate?	N
	4.2 Could measurement or ascertainment of the outcome have differed between intervention groups?	N
	4.3 If N/PN/NI to 4.1 and 4.2: Were outcome assessors aware of the intervention received by study participants?	NI
	4.4 If Y/PY/NI to 4.3: Could assessment of the outcome have been influenced by knowledge of intervention received?	NI

	4.5 If Y/PY/NI to 4.4: Is it likely that assessment of the outcome was influenced by knowledge of intervention received?	PN
Domain 5: Risk of bias in selection of the reported result	5.1 Were the data that produced this result analysed in accordance with a pre-specified analysis plan that was finalized before unblinded outcome data were available for analysis?	NI
	5.2. ... multiple eligible outcome measurements (e.g. scales, definitions, time points)	NI
	5.3 ... multiple eligible analyses of the data?	NI

[25] Chow YWY et al. The effects of Qigong on reducing stress and anxiety and enhancing body–mind well-being

Domain	Signalling questions	Response options
Domain 1: Risk of bias arising from the randomization process	1.1 Was the allocation sequence random?	PY
	1.2 Was the allocation sequence concealed until participants were enrolled and assigned to interventions?	NI
	1.3 Did baseline differences between intervention groups suggest a problem with the randomization process?	N
Domain 2: Risk of bias due to deviations from the intended interventions (effect of assignment to intervention)	2.1. Were participants aware of their assigned intervention during the trial?	PY
	2.2. Were carers and people delivering the interventions aware of participants's assigned intervention during the trial?	PY
	2.3. If Y/PY/NI to 2.1 or 2.2: Were there deviations from the intended intervention that arose because of the trial context?	PN
	2.4 If Y/PY to 2.3: Were these deviations likely to have affected the outcome?	NA
	2.5. If Y/PY/NI to 2.4: Were these deviations from intended intervention balanced between groups?	NA
	2.6 Was an appropriate analysis used to estimate the effect of assignment to intervention?	PY
	2.7 If N/PN/NI to 2.6: Was there potential for a substantial impact (on the result) of the failure to analyse participants in the group to which they were randomized?	NA
Domain 3: Missing outcome data	3.1 Were data for this outcome available for all, or nearly all, participants randomized?	PY
	3.2 If N/PN/NI to 3.1: Is there evidence that the result was not biased by missing outcome data?	NA
	3.3 If N/PN to 3.2: Could missingness in the outcome depend on its true value?	NA
	3.4 If Y/PY/NI to 3.3: Is it likely that missingness in the outcome depended on its true value?	NA
Domain 4: Risk of bias in measurement of the outcome	4.1 Was the method of measuring the outcome inappropriate?	N
	4.2 Could measurement or ascertainment of the outcome have differed between intervention groups?	N
	4.3 If N/PN/NI to 4.1 and 4.2: Were outcome assessors aware of the intervention received by study participants?	NI
	4.4 If Y/PY/NI to 4.3: Could assessment of the outcome have been influenced by knowledge of intervention received?	NI
	4.5 If Y/PY/NI to 4.4: Is it likely that assessment of the outcome was	PN

	influenced by knowledge of intervention received?	
Domain 5: Risk of bias in selection of the reported result	5.1 Were the data that produced this result analysed in accordance with a pre-specified analysis plan that was finalized before unblinded outcome data were available for analysis?	NI
	5.2. ... multiple eligible outcome measurements (e.g. scales, definitions, time points)	NI
	5.3 ... multiple eligible analyses of the data?	NI

[26] Chan ES et al. Biochemical and psychometric evaluation of Self-Healing Qigong as a stress reduction tool among first-year nursing and midwifery students

Domain	Signalling questions	Response options
Domain 1: Risk of bias arising from the randomization process	1.1 Was the allocation sequence random?	Y
	1.2 Was the allocation sequence concealed until participants were enrolled and assigned to interventions?	NI
	1.3 Did baseline differences between intervention groups suggest a problem with the randomization process?	N
Domain 2: Risk of bias due to deviations from the intended interventions (effect of assignment to intervention)	2.1. Were participants aware of their assigned intervention during the trial?	PY
	2.2. Were carers and people delivering the interventions aware of participants's assigned intervention during the trial?	PY
	2.3. If Y/PY/NI to 2.1 or 2.2: Were there deviations from the intended intervention that arose because of the trial context?	Y
	2.4 If Y/PY to 2.3: Were these deviations likely to have affected the outcome?	Y
	2.5. If Y/PY/NI to 2.4: Were these deviations from intended intervention balanced between groups?	PY
	2.6 Was an appropriate analysis used to estimate the effect of assignment to intervention?	PY
	2.7 If N/PN/NI to 2.6: Was there potential for a substantial impact (on the result) of the failure to analyse participants in the group to which they were randomized?	NA
Domain 3: Missing outcome data	3.1 Were data for this outcome available for all, or nearly all, participants randomized?	N
	3.2 If N/PN/NI to 3.1: Is there evidence that the result was not biased by missing outcome data?	N
	3.3 If N/PN to 3.2: Could missingness in the outcome depend on its true value?	PY
	3.4 If Y/PY/NI to 3.3: Is it likely that missingness in the outcome depended on its true value?	NI
Domain 4: Risk of bias in measurement of the outcome	4.1 Was the method of measuring the outcome inappropriate?	N
	4.2 Could measurement or ascertainment of the outcome have differed between intervention groups?	N
	4.3 If N/PN/NI to 4.1 and 4.2: Were outcome assessors aware of the intervention received by study participants?	NI
	4.4 If Y/PY/NI to 4.3: Could assessment of the outcome have been influenced by knowledge of intervention received?	NI

	4.5 If Y/PY/NI to 4.4: Is it likely that assessment of the outcome was influenced by knowledge of intervention received?	PN
Domain 5: Risk of bias in selection of the reported result	5.1 Were the data that produced this result analysed in accordance with a pre-specified analysis plan that was finalized before unblinded outcome data were available for analysis?	PY
	5.2. ... multiple eligible outcome measurements (e.g. scales, definitions, time points)	PY
	5.3 ... multiple eligible analyses of the data?	PY

[27] Hwang EY et al. Effects of a Brief Qigong-based Stress Reduction Program (BQSRP) in a distressed Korean population: a randomized trial

Domain	Signalling questions	Response options
Domain 1: Risk of bias arising from the randomization process	1.1 Was the allocation sequence random?	Y
	1.2 Was the allocation sequence concealed until participants were enrolled and assigned to interventions?	NI
	1.3 Did baseline differences between intervention groups suggest a problem with the randomization process?	N
Domain 2: Risk of bias due to deviations from the intended interventions (effect of assignment to intervention)	2.1. Were participants aware of their assigned intervention during the trial?	PY
	2.2. Were carers and people delivering the interventions aware of participants's assigned intervention during the trial?	PY
	2.3. If Y/PY/NI to 2.1 or 2.2: Were there deviations from the intended intervention that arose because of the trial context?	Y
	2.4 If Y/PY to 2.3: Were these deviations likely to have affected the outcome?	Y
	2.5. If Y/PY/NI to 2.4: Were these deviations from intended intervention balanced between groups?	Y
	2.6 Was an appropriate analysis used to estimate the effect of assignment to intervention?	PY
	2.7 If N/PN/NI to 2.6: Was there potential for a substantial impact (on the result) of the failure to analyse participants in the group to which they were randomized?	NA
Domain 3: Missing outcome data	3.1 Were data for this outcome available for all, or nearly all, participants randomized?	N
	3.2 If N/PN/NI to 3.1: Is there evidence that the result was not biased by missing outcome data?	N
	3.3 If N/PN to 3.2: Could missingness in the outcome depend on its true value?	PY
	3.4 If Y/PY/NI to 3.3: Is it likely that missingness in the outcome depended on its true value?	PY
Domain 4: Risk of bias in measurement of the outcome	4.1 Was the method of measuring the outcome inappropriate?	N
	4.2 Could measurement or ascertainment of the outcome have differed between intervention groups?	N
	4.3 If N/PN/NI to 4.1 and 4.2: Were outcome assessors aware of the intervention received by study participants?	NI
	4.4 If Y/PY/NI to 4.3: Could assessment of the outcome have been influenced by knowledge of intervention received?	NI

	4.5 If Y/PY/NI to 4.4: Is it likely that assessment of the outcome was influenced by knowledge of intervention received?	PN
Domain 5: Risk of bias in selection of the reported result	5.1 Were the data that produced this result analysed in accordance with a pre-specified analysis plan that was finalized before unblinded outcome data were available for analysis?	PY
	5.2. ... multiple eligible outcome measurements (e.g. scales, definitions, time points)	PY
	5.3 ... multiple eligible analyses of the data?	PY

[28] Cheung DST et al. Effect of a Qigong intervention on telomerase activity and mental health in Chinese women survivors of intimate partner violence: A randomized clinical trial

Domain	Signalling questions	Response options
Domain 1: Risk of bias arising from the randomization process	1.1 Was the allocation sequence random?	Y
	1.2 Was the allocation sequence concealed until participants were enrolled and assigned to interventions?	Y
	1.3 Did baseline differences between intervention groups suggest a problem with the randomization process?	N
Domain 2: Risk of bias due to deviations from the intended interventions (effect of assignment to intervention)	2.1. Were participants aware of their assigned intervention during the trial?	PY
	2.2. Were carers and people delivering the interventions aware of participants's assigned intervention during the trial?	PY
	2.3. If Y/PY/NI to 2.1 or 2.2: Were there deviations from the intended intervention that arose because of the trial context?	PN
	2.4 If Y/PY to 2.3: Were these deviations likely to have affected the outcome?	NA
	2.5. If Y/PY/NI to 2.4: Were these deviations from intended intervention balanced between groups?	NA
	2.6 Was an appropriate analysis used to estimate the effect of assignment to intervention?	PY
	2.7 If N/PN/NI to 2.6: Was there potential for a substantial impact (on the result) of the failure to analyse participants in the group to which they were randomized?	NA
Domain 3: Missing outcome data	3.1 Were data for this outcome available for all, or nearly all, participants randomized?	PY
	3.2 If N/PN/NI to 3.1: Is there evidence that the result was not biased by missing outcome data?	NA
	3.3 If N/PN to 3.2: Could missingness in the outcome depend on its true value?	NA
	3.4 If Y/PY/NI to 3.3: Is it likely that missingness in the outcome depended on its true value?	NA
Domain 4: Risk of bias in measurement of the outcome	4.1 Was the method of measuring the outcome inappropriate?	N
	4.2 Could measurement or ascertainment of the outcome have differed between intervention groups?	N
	4.3 If N/PN/NI to 4.1 and 4.2: Were outcome assessors aware of the intervention received by study participants?	N
	4.4 If Y/PY/NI to 4.3: Could assessment of the outcome have been influenced by knowledge of intervention received?	NA

	4.5 If Y/PY/NI to 4.4: Is it likely that assessment of the outcome was influenced by knowledge of intervention received?	NA
Domain 5: Risk of bias in selection of the reported result	5.1 Were the data that produced this result analysed in accordance with a pre-specified analysis plan that was finalized before unblinded outcome data were available for analysis?	PY
	5.2. ... multiple eligible outcome measurements (e.g. scales, definitions, time points)	PY
	5.3 ... multiple eligible analyses of the data?	PY

[29] Ng SM et al. Impact of mind-body intervention on proinflammatory cytokines interleukin 6 and 1β: A three-arm randomized controlled trial for persons with sleep disturbance and depression

**Domain**	**Signalling questions**	**Response options**
Domain 1: Risk of bias arising from the randomization process	1.1 Was the allocation sequence random?	Y
	1.2 Was the allocation sequence concealed until participants were enrolled and assigned to interventions?	Y
	1.3 Did baseline differences between intervention groups suggest a problem with the randomization process?	N
Domain 2: Risk of bias due to deviations from the intended interventions (effect of assignment to intervention)	2.1. Were participants aware of their assigned intervention during the trial?	PY
	2.2. Were carers and people delivering the interventions aware of participants's assigned intervention during the trial?	PY
	2.3. If Y/PY/NI to 2.1 or 2.2: Were there deviations from the intended intervention that arose because of the trial context?	Y
	2.4 If Y/PY to 2.3: Were these deviations likely to have affected the outcome?	Y
	2.5. If Y/PY/NI to 2.4: Were these deviations from intended intervention balanced between groups?	PY
	2.6 Was an appropriate analysis used to estimate the effect of assignment to intervention?	PY
	2.7 If N/PN/NI to 2.6: Was there potential for a substantial impact (on the result) of the failure to analyse participants in the group to which they were randomized?	NA
Domain 3: Missing outcome data	3.1 Were data for this outcome available for all, or nearly all, participants randomized?	N
	3.2 If N/PN/NI to 3.1: Is there evidence that the result was not biased by missing outcome data?	N
	3.3 If N/PN to 3.2: Could missingness in the outcome depend on its true value?	PY
	3.4 If Y/PY/NI to 3.3: Is it likely that missingness in the outcome depended on its true value?	PN
Domain 4: Risk of bias in measurement of the outcome	4.1 Was the method of measuring the outcome inappropriate?	N
	4.2 Could measurement or ascertainment of the outcome have differed between intervention groups?	N
	4.3 If N/PN/NI to 4.1 and 4.2: Were outcome assessors aware of the intervention received by study participants?	NI
	4.4 If Y/PY/NI to 4.3: Could assessment of the outcome have been influenced by knowledge of intervention received?	NI

	4.5 If Y/PY/NI to 4.4: Is it likely that assessment of the outcome was influenced by knowledge of intervention received?	PN
Domain 5: Risk of bias in selection of the reported result	5.1 Were the data that produced this result analysed in accordance with a pre-specified analysis plan that was finalized before unblinded outcome data were available for analysis?	PY
	5.2. ... multiple eligible outcome measurements (e.g. scales, definitions, time points)	PY
	5.3 ... multiple eligible analyses of the data?	PY

[30] Zhang S et al. The effectiveness of traditional Chinese Yijinjing Qigong exercise for patients with knee osteoarthritis on pain, dysfunction, and mood disorder: A pilot randomized controlled trial

**Domain**	**Signalling questions**	**Response options**
Domain 1: Risk of bias arising from the randomization process	1.1 Was the allocation sequence random?	Y
	1.2 Was the allocation sequence concealed until participants were enrolled and assigned to interventions?	Y
	1.3 Did baseline differences between intervention groups suggest a problem with the randomization process?	N
Domain 2: Risk of bias due to deviations from the intended interventions (effect of assignment to intervention)	2.1. Were participants aware of their assigned intervention during the trial?	PY
	2.2. Were carers and people delivering the interventions aware of participants's assigned intervention during the trial?	PY
	2.3. If Y/PY/NI to 2.1 or 2.2: Were there deviations from the intended intervention that arose because of the trial context?	PN
	2.4 If Y/PY to 2.3: Were these deviations likely to have affected the outcome?	NA
	2.5. If Y/PY/NI to 2.4: Were these deviations from intended intervention balanced between groups?	NA
	2.6 Was an appropriate analysis used to estimate the effect of assignment to intervention?	PY
	2.7 If N/PN/NI to 2.6: Was there potential for a substantial impact (on the result) of the failure to analyse participants in the group to which they were randomized?	NA
Domain 3: Missing outcome data	3.1 Were data for this outcome available for all, or nearly all, participants randomized?	PY
	3.2 If N/PN/NI to 3.1: Is there evidence that the result was not biased by missing outcome data?	NA
	3.3 If N/PN to 3.2: Could missingness in the outcome depend on its true value?	NA
	3.4 If Y/PY/NI to 3.3: Is it likely that missingness in the outcome depended on its true value?	NA
Domain 4: Risk of bias in measurement of the outcome	4.1 Was the method of measuring the outcome inappropriate?	N
	4.2 Could measurement or ascertainment of the outcome have differed between intervention groups?	N
	4.3 If N/PN/NI to 4.1 and 4.2: Were outcome assessors aware of the intervention received by study participants?	N
	4.4 If Y/PY/NI to 4.3: Could assessment of the outcome have been influenced by knowledge of intervention received?	N

	4.5 If Y/PY/NI to 4.4: Is it likely that assessment of the outcome was influenced by knowledge of intervention received?	NA
Domain 5: Risk of bias in selection of the reported result	5.1 Were the data that produced this result analysed in accordance with a pre-specified analysis plan that was finalized before unblinded outcome data were available for analysis?	PY
	5.2. ... multiple eligible outcome measurements (e.g. scales, definitions, time points)	PY
	5.3 ... multiple eligible analyses of the data?	PY

[31] Wu F et al. Tracking studies on the effects of Qi Gong fitness on stress, anxiety, and depression among underprivileged working youth

**Domain**	**Signalling questions**	**Response options**
Domain 1: Risk of bias arising from the randomization process	1.1 Was the allocation sequence random?	Y
	1.2 Was the allocation sequence concealed until participants were enrolled and assigned to interventions?	NI
	1.3 Did baseline differences between intervention groups suggest a problem with the randomization process?	N
Domain 2: Risk of bias due to deviations from the intended interventions (effect of assignment to intervention)	2.1. Were participants aware of their assigned intervention during the trial?	PY
	2.2. Were carers and people delivering the interventions aware of participants's assigned intervention during the trial?	PY
	2.3. If Y/PY/NI to 2.1 or 2.2: Were there deviations from the intended intervention that arose because of the trial context?	N
	2.4 If Y/PY to 2.3: Were these deviations likely to have affected the outcome?	NA
	2.5. If Y/PY/NI to 2.4: Were these deviations from intended intervention balanced between groups?	NA
	2.6 Was an appropriate analysis used to estimate the effect of assignment to intervention?	PY
	2.7 If N/PN/NI to 2.6: Was there potential for a substantial impact (on the result) of the failure to analyse participants in the group to which they were randomized?	NA
Domain 3: Missing outcome data	3.1 Were data for this outcome available for all, or nearly all, participants randomized?	PY
	3.2 If N/PN/NI to 3.1: Is there evidence that the result was not biased by missing outcome data?	NA
	3.3 If N/PN to 3.2: Could missingness in the outcome depend on its true value?	NA
	3.4 If Y/PY/NI to 3.3: Is it likely that missingness in the outcome depended on its true value?	NA
Domain 4: Risk of bias in measurement of the outcome	4.1 Was the method of measuring the outcome inappropriate?	N
	4.2 Could measurement or ascertainment of the outcome have differed between intervention groups?	Y
	4.3 If N/PN/NI to 4.1 and 4.2: Were outcome assessors aware of the intervention received by study participants?	NA
	4.4 If Y/PY/NI to 4.3: Could assessment of the outcome have been influenced by knowledge of intervention received?	NA

	4.5 If Y/PY/NI to 4.4: Is it likely that assessment of the outcome was influenced by knowledge of intervention received?	NA
Domain 5: Risk of bias in selection of the reported result	5.1 Were the data that produced this result analysed in accordance with a pre-specified analysis plan that was finalized before unblinded outcome data were available for analysis?	NI
	5.2. ... multiple eligible outcome measurements (e.g. scales, definitions, time points)	NI
	5.3 ... multiple eligible analyses of the data?	NI
